# Research of the Potential Vaginal Microbiome Biomarkers for High-Grade Squamous Intraepithelial Lesion

**DOI:** 10.3389/fmed.2021.565001

**Published:** 2021-09-21

**Authors:** Xiaopei Chao, Lan Wang, Shu Wang, Jinghe Lang, Xianjie Tan, Qingbo Fan, Honghui Shi

**Affiliations:** ^1^Department of Obstetrics and Gynecology, Peking Union Medical College Hospital, Chinese Academy of Medical Sciences, Peking Union Medical College, Beijing, China; ^2^National Clinical Research Center for Obstetric and Gynecologic Diseases, Beijing, China; ^3^Department of Obstetrics and Gynecology, Hebei Yanda Hospital, Hebei, China

**Keywords:** vaginal microbiome, squamous intraepithelial lesion, 16S rRNA, biomarker, diagnosis

## Abstract

Vaginal microbiome may have a role in HPV infection and cervical neoplasm. To explore potential vaginal microbiome biomarkers for high-grade squamous intraepithelial lesion (HSIL), and to find the best scheme to facilitate the current cervical cancer screening strategy. This study enrolled 272 women, including 83 confirmed with HSIL, 86 with HPV infection but without cervical neoplasm, and 103 without HPV infection as controls. Vaginal microbiome composition was determined by sequencing of barcoded 16S rDNA gene fragments (V4) on Illumina HiSeq2500. The relative increasing abundance of *Stenotrophomonas, Streptococcus*, and *Pseudomonas*, and a concomitant paucity of *Dialister, unidentified Prevotellaceae, Faecalibacterium, Bifidobacterium*, and *Bacteroides*, were related with HSIL, which can be used to predict the development of HISL in high-risk HPV infected patients. The relative abundance of *Stenotrophomonas* being over 0.0090387%, or *Faecalibacterium* being under 0.01420015%, or *Bifidobacterium* being under 0.0116183% maybe a good predictor for HSIL for those infected with HPV 16 and/or 18. The relative abundance of *Stenotrophomonas* being over 0.01549105%, or *Streptococcus* being over 0.48409585%, or *Bacteroides* being under 0.0296912% maybe a good predictor for HSIL for those infected with the 12 other high-risk types of HPV with concurrent abnormal TCT results. This study revealed that potential vaginal microbiome biomarkers may relate to HSIL, and can facilitate the cervical cancer screening.

## Introduction

Human Papillomavirus (HPV) is one of the most common causes of sexually transmitted diseases (STDs) in women around the world (1). The incidence of HPV infection is common throughout life (>80% in sexually active people), co-infections of multiple HPV types are likely to occur in approximately more than 30% of HPV patients (1). However, the incidence of HPV-related diseases is relatively lower (2). 10–20% of HPV infections persist latently (3), and only 0.3–1.2% of the initial infections will eventually progress to invasive cervical cancer. Persistent high-risk HPV (hrHPV) infection does not always result in cervical intraepithelial neoplasm/cancer, and other exposures are thought to play important roles, such as vaginal microbiota (VMB) dysbiosis.

The vaginal ecosystem exists as a finely tuned balance between microorganisms and the host. Modern next-generation sequencing-based characterization of the VMB has provided a more in-depth and detailed composition of the microbiota. There is emerging evidence that VMB may play a crucial role in HPV induced cervical lesions (4–6) and is related to protection against dysbiosis and HPV infection (7, 8). It provides the evidence that sexually active women with vaginal dysbiosis are at increased risk of developing associated premalignant and malignant cervical disease (6). Cervicovaginal dysbiosis states (which could be caused by multiple factors in addition to HPV infection or neoplastic cells) reduce cervicovaginal barrier function (9) and alter metabolic profiles (10), and these may, in turn, facilitate HPV acquisition and cervical intraepithelial neoplasm/cancer development, respectively.

Cervical cytology and HPV tests are widely used for cervical cancer screening and thus early detection of underlying disease. However, although the current screening strategy is highly sensitive for high-grade cervical neoplasm, it holds a limited specificity. Therefore, the objective of our current pilot study is to explore the most closed specific compositions of the VMB (defined by molecular techniques) associated with high-grade squamous intraepithelial lesion (HSIL), and thus facilitate the current screening strategy by decreasing the proportion of cases receiving invasive examination and overtreatment.

## Materials and Methods

### Ethics

Ethical approval was obtained from the Ethics Committee of Peking Union Medical College Hospital (PUMCH), Beijing, China (No. JS-1634, registered on July 24, 2018). All experiments were performed in accordance with relevant guidelines and regulations. The registration No. on clinicaltrials.gov is NCT03548740. Written informed consent was obtained from all participants.

### Study Design

**Figure 1:**
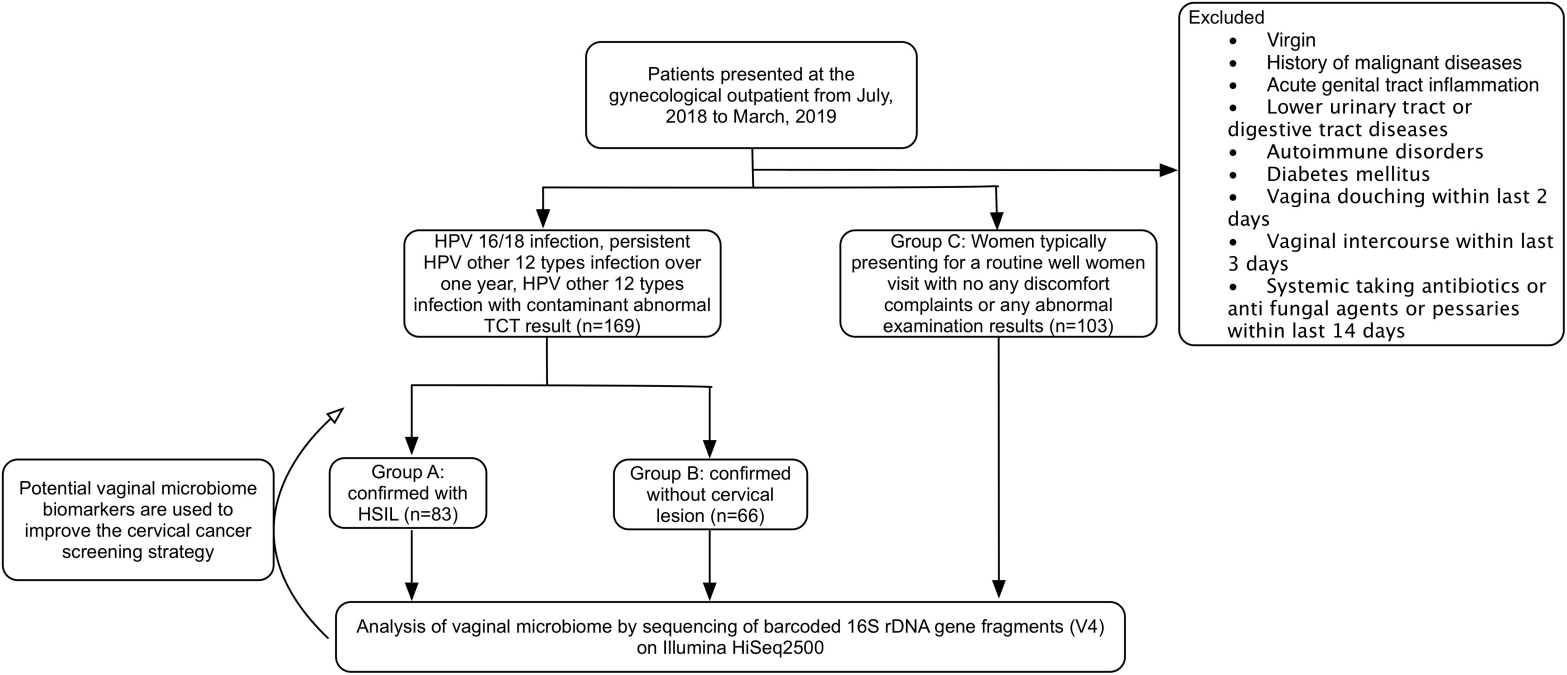
CONSORT flow diagram of the study. HPV, human papilloma virus; HSIL, high-grade squamous intraepithelial lesion; TCT, ThinPrep^®^ Pap testing.

This prospective observational cohort study was implemented in a tertiary teaching hospital. The sample size was referred to the previous reported study. According to the results of HPV test and pathology of cervical biopsy, the participants were divided into three groups. Group A: 83 cases with HPV infection confirmed with HSIL by cervical biopsy (patients infected with HPV type 16 and/or 18; infected with the other 12 types of HR-HPV for more than 1 year; infected with the other 12 types of high-risk HPV and abnormal cervical cytology). Group B: 86 cases with HPV infection but confirmed without cervical neoplasm (LSIL were excluded) (patients infected with HPV type 16 and/or 18; infected with the other 12 types of HR-HPV for more than 1 year; infected with the other 12 types of HR-HPV and abnormal cervical cytology). Group C: 103 cases without HPV infection or abnormal TCT result (the participants came to visit just for routine physical examination, and their test results of the current HPV and TCT status were negative). All the patients enrolled haven't been treated with physiotherapy such as laser therapy, cryotherapy, or surgical treatment like loop electrosurgical excision or cold knife conization. The flow diagram of this study is showed in Figure 1.

### Study Population

The participants engaged in this research were those who visited the department of Obstetrics & Gynecology of PUMCH between July 2018 and March 2019. All of the participants enrolled are women presenting for cervical cancer screening. Inclusion criteria: Those aged 20 to 72 years old, having had vaginal intercourse for more than 3 years, and aren't in menstrual, pregnancy or puerperium period. Exclusion criteria: Those who are virgin, having had total or subtotal hysterectomy, or patients who were diagnosed with acute genital tract inflammation. Women who are HIV positive, have autoimmune disorders, or have a history of malignant tumors are also excluded. At the same time, all the participants should meet the following requirements: no vagina douching within the last 2 days, no vaginal intercourse within the last 3 days, no systemic application of antifungal agents, antibiotics or pessaries within the last 14 days before sampling.

### Specimen Collection

A sterile, disposable speculum was inserted without lubricant, and a sterile swab sample was taken from the posterior vaginal fornix and stored immediately under −80°C for DNA extraction. At the same time, each patient was given a liquid Pap test with ThinPrep^®^ Pap testing (Hologic, Inc., MA) and DNA capture via the Cobas^®^ 4800 System HPV Genotyping Test (Roche Molecular Diagnostics, CA) which is based on real-time qualitative PCR (RQ-PCR).

DNA extraction and the amplification of bacterial 16S rRNA V4 gene region and Illumina sequence were shown in the Supplementary Material.

### Data Analysis

Statistical analysis of the clinical data was performed using the SPSS 23.0 software (SPSS Inc., Chicago, IL, USA). Continuous variables were analyzed with rank sum test, and categorical variables were analyzed with Chi-Square Test. *P* < 0.05 was interpreted to be statistically significant.

## Results

### Sociodemographic and Clinical Baseline Characteristics

The baseline characteristics were generally similar within the three groups. The mean ages of the three groups were 38.34 ± 10.18, 39.00 ± 9.28, and 39.35 ± 9.43 years old, respectively. There was no significant difference within the three groups regarding age (*P* = 0.773), Gravidity (*P* = 0.057), parity (*P* = 0.541), phase of menstrual cycle (*P* = 0.177) and the method of contraception (*P* = 0.489; Table 1). Among the 169 cases confirmed with HPV infection, there is a total of 63 cases infected with HPV type 16 and/or 18, and 90 cases infected with the other 12 types of HR-HPV. Among the 63 cases infected with HPV type 16 and/or 18, 48 were confirmed to have HSIL, while 15 cases were confirmed to have no cervical lesions. Only two cases were confirmed with persistent infection of the other 12 types of HR-HPV, with one case each in group A and group B. Besides, 40 patients were infected with the other 12 types of HR-HPV with concurrent abnormal TCT results, and 16 cases were diagnosed with HSIL while 24 cases were without cervical lesion.

**Table 1:** Patients' sociodemographic and clinical baseline characteristics.

**Characteristics**	**Group A** **CIN2+** **(***n*** = 83)**	**Group B** **HPV infection without** **cervical neoplasia** **(***n*** = 86)**	**Group C** **HPV negative** **(***n*** = 103)**	**Total** **(***n*** = 272)**	***P*** **-value**
Age, years					0.773
Mean ± SD	38.34 ± 10.18	39.00 ± 9.28	39.35 ± 9.43	38.93 ± 9.59	
Parity, n/N (%)				0.493	
Nulliparous	27/83 (32.53)	27/86 (31.40)	26/103 (25.24)	80/272 (29.41)	
Parous	56/83 (67.47)	59/86 (68.60)	77/103 (74.76)	192/272 (70.59)	
Situation of fertility					
Gravidity	2.08 ± 1.68	1.77 ± 1.28	1.58 ± 1.30	1.79 ± 1.43	0.057
Parity	1.00 ± 0.90	0.87 ± 0.72	0.91 ± 0.69	0.93 ± 0.77	0.541
Phase of menstrual cycle, n/N (%)				0.177	
Follicular	43/83 (51.81)	34/86 (39.53)	36/103 (34.95)	113/272 (41.54)	
Luteal	24/83 (28.92)	33/86 (38.37)	43/103 (41.75)	100/272 (36.76)	
Postmenopausal	6/83 (7.23)	13/86 (15.12)	13/103 (12.62)	32/272 (11.76)	
NA	10/83 (12.05)	6/86 (6.98)	11/103 (10.68)	27/272 (9.23)	
Contraception, n/N (%)					0.489
Nil	65/83 (78.31)	68/86 (79.07)	72/103 (69.90)	205/272 (75.37)	
Condoms	14/83 (16.87)	12/86 (13.95)	24/103 (23.30)	50/272 (18.38)	
IUD	4/83 (4.82)	6/86 (6.98)	7/103 (6.80)	17/272 (6.25)	
HPV status, n/N (%)					
HPV 16/18 positive	48/83 (57.83)	15/86 (17.44)			
HPV other12 positive	22/83 (26.5)	68/86 (79.07)			
NA	13/83 (15.66)	3/86 (3.49)			

*IUD, Intrauterine device; SD, standard deviation. P-value is calculated using Pearson Chi-Square and Rank sum test*.

### Microbiome Community Diversity

#### Identification of Vaginal Microbiome

**Figure 2:**
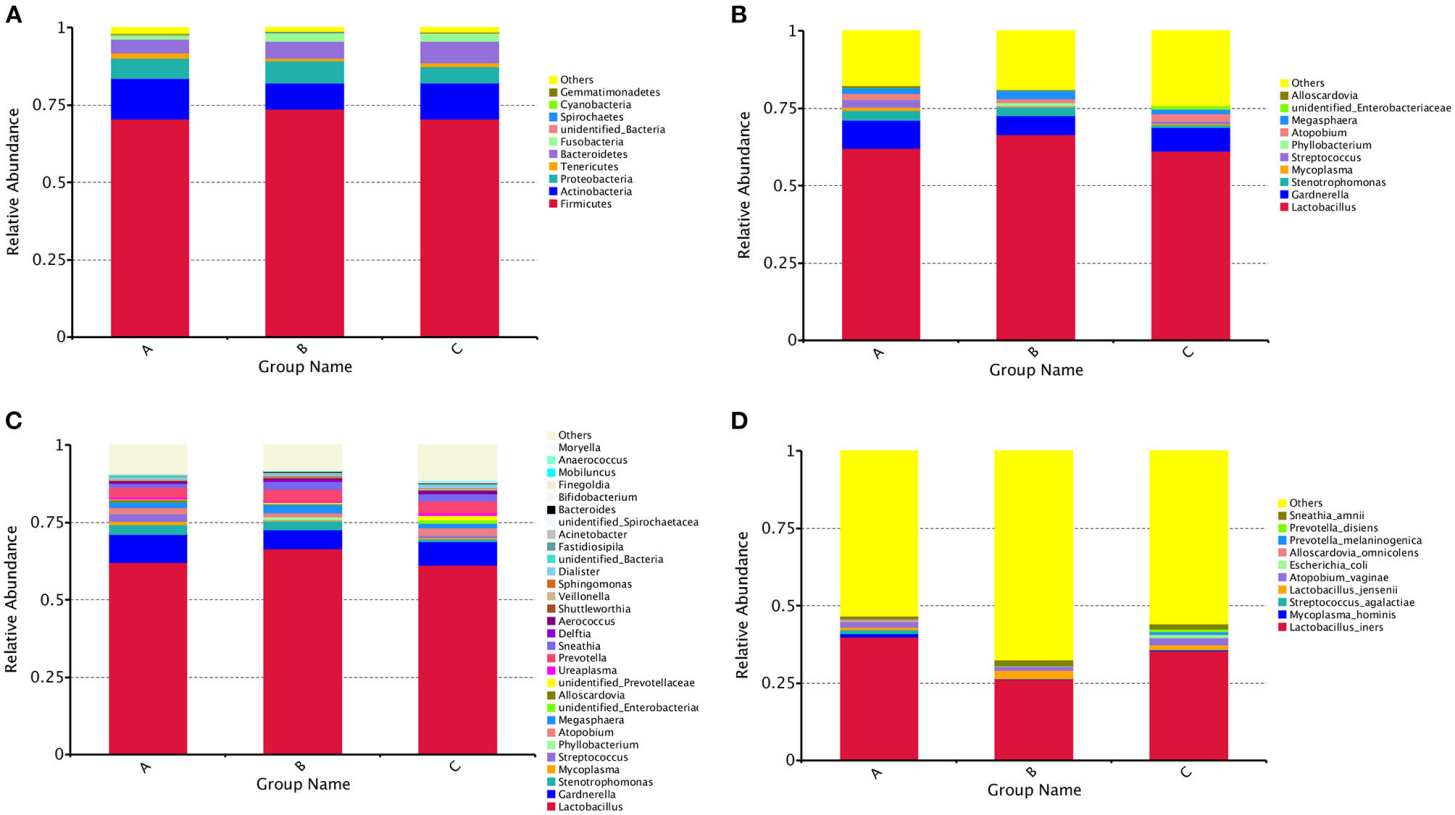
The community composition of vaginal bacterial in three groups. **(A)** Bar chart of relative abundance of top 10 phyla of each group. **(B)** Bar chart of relative abundance of top 10 genera of each group. **(C)** Bar chart of relative abundance of top 30 genera of each group. **(D)** Bar chart of relative abundance of top 10 species of each group.

A total of 55 phyla, 1,217 genera and 1,211 species were detected. The distribution of the vaginal bacteria at different levels are shown in Figure 2.

#### The Structure of the Vaginal Microbiome Within the Three Groups

**Figure 3:**
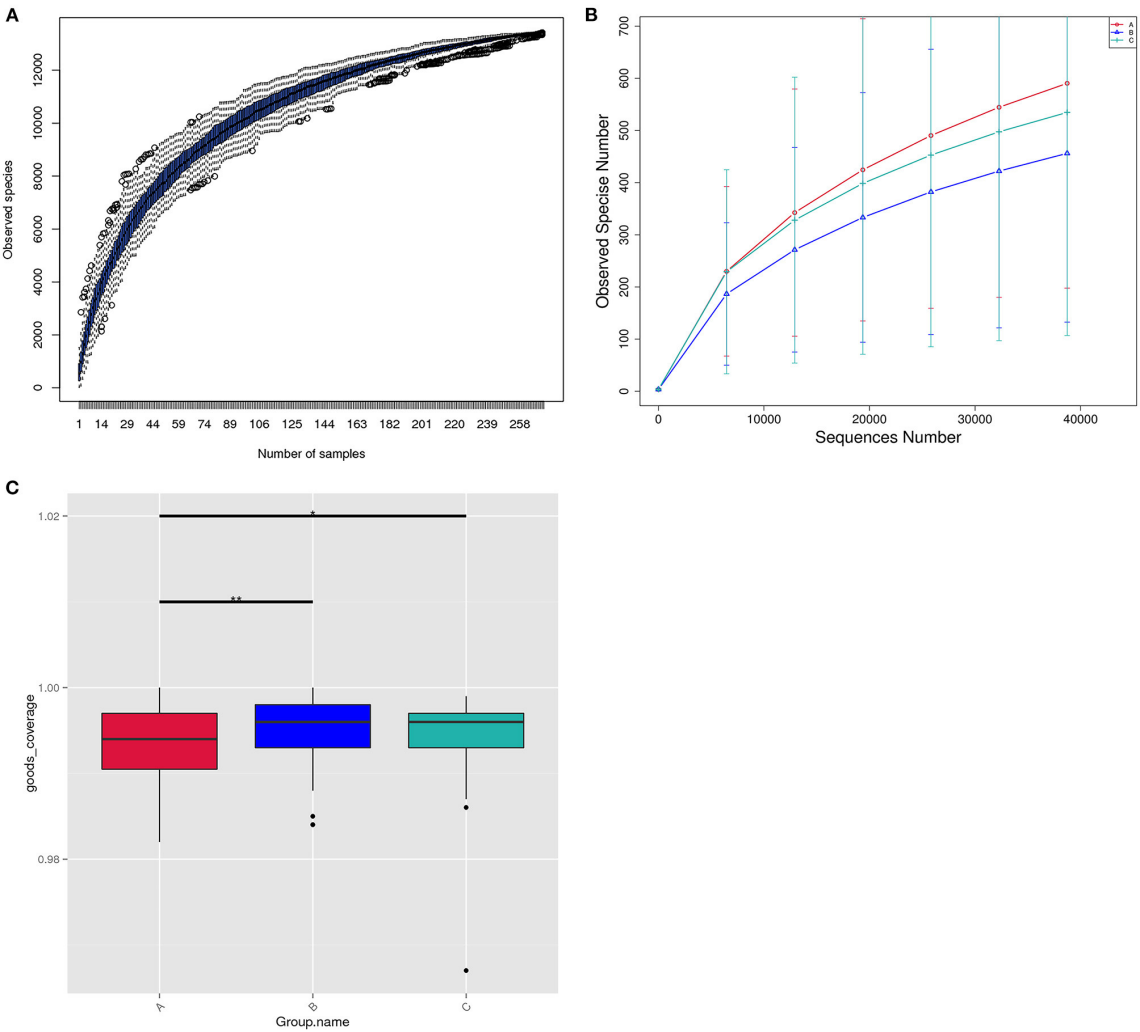
The structure of the vaginal microbiome within three groups. **(A)** Species accumulation boxplot of all the 272 samples in three groups. **(B)** Rarefaction curve of vaginal microbiome diversity in three groups, and error bars represent standard deviation. Alpha diversity analyses revealed Observed Species differences within three groups. **(C)** Bar chart of microbiota diversity for each group.

Figure 3A showed that the species diversity increased along with the increasing of the sample size, and suggested that the sample size was adequate for analysis. From the rarefaction curve (Figure 3B), we can see that those from group A had the highest microbiome diversity, followed by group C, and group B had the lowest diversity. The microbiome diversity was much richer in group A than B, C (A vs. B, *P* = 0.0065; A vs. C, *P* = 0.0253; B vs. C, *P* = 0.5359; Figure 3C).

#### Identification of Vaginal Microbiome Composition Within the Three Groups

**Figure 4:**
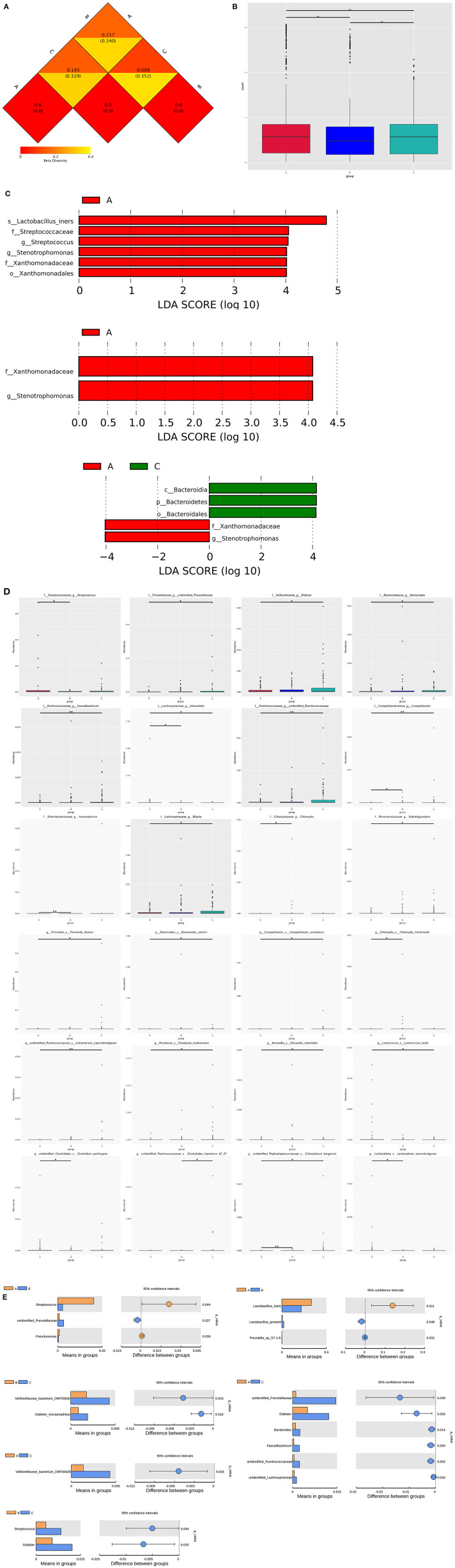
Vaginal microbiome composition markers within three groups. **(A)** Distance Matrix Heatmap based on the weighted unifrac distance showing the intergroup difference within three groups. The number in the graph is the difference coefficient between two samples. The smaller the difference coefficient is, the smaller the difference of microbiome diversity is. **(B)** Bar chart of the comparisons of vaginal microbiota for intergroup difference analysis. **(C)** LEfSe identifies bacterial clades that are differentially abundant within three groups. Clades in this graph were both statistically significant (*P* < 0.05) and had an LDA score >±4, considered a significant effect size. Prefixes represent abbreviations for taxonomic rank of each taxa: phylum (p_), class (c_), order (o_), family (f_), genus (g_), species (s_). **(D)** Significantly different abundance of bacterial clades within groups by MetaStat analysis. **(E)** Significantly different abundance of genera and species within groups by *t*-test analysis.

Distance Matrix Heatmap based on the weighted unifrac distance revealed that the vaginal microbiome's difference between group A and B was the greatest, and that between group B and C was the smallest (Figure 4A). The vaginal composition were significantly different within the three groups (A vs. B, A vs. C, B vs. C, *P* < 0.05; Figure 4B).

At the genus level of bacteria, the relative abundance of *Stenotrophomonas* gradient decreased successively according to the order of group A, B, and C and statistical difference exists within the three groups. The relative abundance of several genera gradient increased drastically according to the order of group A, B, and C with significant difference, including *Dialister, Mobiluncus, Faecalibacterium, unidentified Prevotellaceae, unidentified Ruminococcaceae* and *unidentified Lachnospiraceae*. Besides, the relative abundance of *Delftia* and *Bacteroides* were the highest in group B, and *Bifidobacterium* was the highest in group C. Group A had the highest relative abundance of *Streptococcus* and *Pseudomonas* (Figures 4C–E and Tables 2, 3).

**Table 2:** The statistically different genera and species within three groups.

	**Relative abundance**			
**Taxa**	**Group A**	**Group B**	***Q*** **-value**	**Method of analysis**
*g_Streptococcus*	0.02433	0.003354	*0.0126152505689353*	Metastat analysis
			***P*** **-value**	
*g_Pseudomonas*	0.001136	0.000561	0.039	t-test analysis
*g_Lactobacillus; s_Lactobacillus_iners*	0.398547	0.26135	0.0108516346283056	
*g_Lactobacillus; s_Lactobacillus_jensenii*	0.008099	0.026327	0.049470925029558	
*g_unidentified_Prevotellaceae; s_Prevotella_sp_S7-1-8;*	0.0003	0.002227	0.0306323059117605	
			***Kruskal-Wallis test P*** **-value**	
*g_Stenotrophomonas*	0.03043	0.026599	*0.0297206112644/LDA = 4.01923299368*	LEfSe analysis
*g_Delftia*	0.002311	0.00627	*0.0425357324452*	
*g_Lactobacillus. s_Lactobacillus_intestinalis*	0.001403	0.000984	*0.0377816191524*	
*g_Streptococcus. s_Streptococcus_agalactiae*	0.012113	0.000705	*0.02937052722*	
*g_Sneathia. s_Sneathia_amnii*	0.0079	0.017207	*0.0384594169478*	
*g_Delftia. s_Delftia_tsuruhatensis*	0.002311	0.00627	*0.0425357324452*	
**Taxa**	**Group A**	**Group C**	***Q*** **-value**	
*g_unidentified_Prevotellaceae*	0.00112	0.01459	*0.0165174192323514*	Metastat analysis
*g_Dialister*	0.00503	0.01219	*0.047162140185396*	
*g_Bacteroides*	0.000999	0.002484	*0.0255626726214962*	
*g_Faecalibacterium*	0.000439	0.002269	*0.00664908861630173*	
*g_unidentified_Ruminococcaceae*	0.000512	0.002368	*0.00664908861630173*	
			***P*** **-value**	
*g_unidentified_Lachnospiraceae*	0.000597	0.001355	*0.016*	t-test analysis
*g_Dialister; s_Veillonellaceae_bacterium_DNF00626*	0.002437	0.007826	*0.015918456426067*	
			***Kruskal-Wallis test P*** **-value**	
*g_Stenotrophomonas*	0.03043	0.009992	*0.0170377050151/LDA = 4.03395504567*	LEfSe analysis
*g_Delftia*	0.002311	0.001254	*0.000862448621444*	
*g_Bifidobacterium*	0.001164	0.002841	*0.00161970367599*	
*g_Mobiluncus*	0.000071	0.001019	*0.0297397041713*	
*g_Lactobacillus. s_Lactobacillus_reuteri*	0.003339	0.001218	0.0334917506149	
*g_Lactobacillus. s_Lactobacillus_intestinalis*	0.001403	0.000963	0.00822711615751	
*g_Streptococcus. s_Streptococcus_agalactiae*	0.012113	0.001076	0.0339276686372	
*g_unidentified_Prevotellaceae. s_Prevotella_sp_S7_1_8*	0.0003	0.001714	0.0200107492257	
*g_Prevotella. s_Prevotella_disiens*	0.000149	0.007642	0.00168221295205	
*g_Sneathia. s_Sneathia_amnii*	0.0079	0.01751	0.00610185979889	
*g_Delftia. s_Delftia_tsuruhatensis*	0.002311	0.001254	0.000862448621444	
**Taxa**	**Group B**	**Group C**	***P*** **-value**	
*g_Dialister*	0.005457	0.01219	0.0254409491678661	t-test analysis
*g_Dialister; s_Veillonellaceae_bacterium_DNF00626*	0.003211	0.007826	0.0429323293790955	
*g_Dialister; s_Dialister_micraerophilus*	0.001564	0.003451	0.0156376539971847	
			***Kruskal-Wallis test P*** **-value**	
*g_Prevotella. s_Prevotella_disiens*	0.000377	0.007642	0.0233918186883	LEfSe analysis
*g_Sphingomonas. s_Sphingomonas_leidyi*	0.002984	0.000323	0.0407776966595	
*g_Moryella. s_Moryella_sp_KHD1*	0.000206	0.001106	0.0445293042836	

*All of the significant different genera and species have relative abundance more than 0.001*.

**Table 3:** The statistically different genus and species within three groups by LEfSe analysis.

	**Relative abundance**				
**Taxa (genus)**	**Group A**	**Group B**	**Group C**	***Kruskal-Wallis test P*** **-value**	**Method of analysis**
*g__Stenotrophomonas*	0.03043	0.026599	0.009992	*0.0322394503223*	LEfSe analysis
*g__Delftia*	0.002311	0.00627	0.001254	*0.00463236955053*	
*g__Bifidobacterium*	0.001164	0.000953	0.002841	*0.00617562607026*	
**Taxa (species)**	**Group A**	**Group B**	**Group C**	***Kruskal-Wallis test P*** **-value**	
*g__Lactobacillus. s__Lactobacillus_intestinalis*	0.001403	0.000984	0.000963	0.018991110686	LEfSe analysis
*g__Streptococcus. s__Streptococcus_agalactiae*	0.012113	0.000705	0.001076	0.0467837765184	
*g__Prevotella. s__Prevotella_disiens*	0.000149	0.000377	0.007642	0.00392649931071	
*g__Sneathia. s__Sneathia_amnii*	0.0079	0.017207	0.01751	0.0200923859974	
*g__Delftia. s__Delftia_tsuruhatensis*	0.002311	0.00627	0.001254	0.00463236955053	
*g__Sphingomonas. s__Sphingomonas_leidyi*	0.000368	0.002984	0.000323	0.0203638415012	

*All of the significant different genera and species have relative abundance more than 0.001*.

At the species level of bacteria, the relative abundance of *Lactobacillus intestinalis* and *Lactobacillus reuteri* gradient decreased with significant difference according to the order of group A, B, and C. The relative abundance of *Prevotella disiens, Sneathia amnii* and *Veillonellaceae bacterium DNF00626* gradient increased according to the order of group A, B, and C with significant difference. The relative abundance of *Streptococcus_agalactiae* and *Lactobacillus_iners* were the highest in group A, while those of *Dialister_micraerophilus* and *Moryella_sp_KHD1* were the highest in group C. Besides, the relative abundance of several species were the highest in group B, including *Delftia_tsuruhatensis, Sphingomonas_leidyi, Prevotella_sp_S7-1-8*, and *Lactobacillus_jensenii* (Figures 4C–E and Tables 2, 3).

### The Diagnostic Efficacy of the Vaginal Microbiome

We performed the Receiver Operating Characteristic (ROC) Curve to find the cut-off relative abundance of potential microbiome biomarkers to help avoid unnecessary invasive cervical biopsy. Among the 63 cases infected with HPV type 16 and/or 18, 15 cases were confirmed to have no cervical lesions by cervical biopsy. The positive predictive value (PPV) of HSIL can reach from 76.19 to 82.54% with the relative abundance of *Stenotrophomonas* being over 0.0090387%, or *Faecalibacterium* being under 0.01420015%, or *Bifidobacterium* being under 0.0116183%. That is, 4 cases could avoid the invasive biopsy (Table 4).

**Table 4:** Accuracy of different strategy in screening patients with HSIL.

	**HSIL (cases)**		**PPV (%)**
**Screening method**	**Yes**	**No**	
HPV 16 and/or 18 positive			
Met	48	15	76.19
HPV 16 and/or 18 positive plus vaginal microbiome			
Met	52	11	82.54
Incident infection of HPV other 12 types with abnormal TCT result ^*^			
Met	16	24	40.00
Incident infection of HPV other 12 types with abnormal TCT result plus vaginal microbiome			
Met	27	13	67.50

* *Normal TCT result is defined as “no intraepithelial lesions or malignant cells (NILM)”, while abnormal TCT result includes “atypical squamous cells of unknown significance (ASCUS)”, “lower-grade squamous intraepithelial lesions (LSIL)”, “high-grade squamous intraepithelial lesions (HSIL)”, and ”atypical squamous cells: cannot exclude high-grade squamous intraepithelial lesions (ASC-H)”*.

*HPV, human papillomavirus; NPV, negative predictive value; PPV, positive predictive value; Sen., sensitivity; Spe., specificity; TCT, ThinPrep cytology test*.

Besides, there were 40 patients who were infected with the other 12 types of high-risk HPV with concurrent abnormal TCT results, among which 16 cases were diagnosed with HSIL while 24 were without cervical lesion. The PPV can reach from 40.00 to 67.50% with the relative abundance of *Stenotrophomonas* being over 0.01549105%, or *Streptococcus* being over 0.48409585%, or *Bacteroides* being under 0.0296912%. That is, 11 cases could avoid the invasive biopsy (Table 4).

## Discussion

Data suggest that long-term persistence of HR-HPV may not always result in HSIL (11). Accumulating evidence suggests that both HPV and bacterial dysbiosis might play a significant role in malignant transformation. Nonetheless, our knowledge about the interactions between HPV infection and the bacterial microbiota and its impact on human health is still rudimentary (12). It has been reported that an increasing VMB diversity is related to HPV acquisition and persistence, as well as development of cervical intraepithelial neoplasm and cervical cancer (4, 13–16). Laniewski et al. (17) also pointed out that high microbiome diversity and *Lactobacillus* depletion correlates with the severity of cervical neoplasm. It is those with the highest diversity of VM having the greatest instability (i.e., transition from one state to another) (18). In our pilot study, we also revealed that those confirmed with HSIL had the highest VMB diversity, which is consistent with the previous studies.

Besides the finding regarding composition diversity, we also found several potential biomarkers related to cervical neoplasm. At genus level, patients confirmed with HSIL had a vaginal microbial pattern characterized by high abundance of *Stenotrophomonas, Streptococcus*, and *Pseudomonas*, as well as concomitant paucity of *Dialister, unidentified_Prevotellaceae, Faecalibacterium, Bifidobacterium*, and *Bacteroides*. Numerous epidemiological studies have shown associations between the non-*Lactobacillus*-dominant (NLD) cervicovaginal microenvironment and HPV infection, development of precancerous dysplasia and progression to cervical cancer (12, 17, 19). Multiple bacterial taxa have been identified to be associated with cervical neoplasia (*Sneathia, Atopobium, Parvimonas, Fusobacterium, Anaerococcus, Peptostreptococcus*) (13, 14, 17). It has also been reported that four bacterial genera being in low abundance (Bifidobacterium, *Moryella, Schlegella*, and *Aerococcus*) and one being in high abundance (*Gardnerella*) is associated with cervical lesions (20). Patients with HPV infection progressed into cervical intraepithelial neoplasia (CIN) are usually colonized by *Sneathia*, while in women with invasive cervical cancer, *Fusobacterium* was the most common type of microorganism (14). Besides, some studies identified *Sneathia spp*. to be associated with HPV detection and/or cervical neoplasm (13, 14, 19). *Fusobacterium* may be oncogenic and might promote the development of dysplasia (13, 14). Thus, *Sneathia* predominates in women with CIN, but not ICC (14). *Faecalibacterium*, one of the immune-modulating bacterial genera (21, 22), could play a role in the progression of disease in later stages of infection.

Besides the findings at genera level, this study also revealed results of great importance at species level. In patients confirmed with HSIL, the relative abundance of 11 types of bacteria are significantly different from the other groups, including the highest abundance of *Lactobacillus intestinalis, Lactobacillus reuteri, Lactobacillus iners, Streptococcus agalactiae*, and a concomitant paucity of *Lactobacillus jensenii, Dialister micraerophilus, Moryella sp KHD1, Prevotella disiens, Sneathia amnii, Veillonellaceae bacterium DNF00626*, and *Prevotella sp S7-1-8*. A study conducted with Korean women also showed that a cervical microbial pattern characterized by high abundance of *Atopobium vagiane, Lactobacillus iners*, and *Gardnerella vaginalis* and a concomitant paucity of *Lactobacillus crispatus* causes an almost 6× higher risk of cervical neoplasm (15, 23). Synergistic effect of this microbial pattern and oncogenic HPV infection leads to a very high risk (odds ratio of 34.1) of CIN (23). Women with HSIL had significantly higher abundance of *Sneathia sanguinegens, Anaerococcus tetradius* and *Peptostreptococcus anaerobius* and lower abundance of *Lactobacillus. jensenii* compared to those with LSIL (5, 13). *Sneathia spp., Megasphaera elsdenii* and *Shuttleworhia satelles* were the most representative in the SIL cases (14). A recent systematic review and meta-analysis showed that bacterial vaginosis is associated with increased risks of incident HR-HPV (relative risk 1.33), HR-HPV persistent (1.18), and CIN/cancer (2.01) (6). It is also reported that there is a positive association of *Prevotella timonensis, Prevotella amnii*, and *Prevotella micra* with CIN3 lesions, taxa previously associated with bacterial vaginosis (24). Although it is unknown whether a *Lactobacillus*-dominated VMB protects women from adverse reproductive health outcomes, not all *Lactobacillus* are necessarily stable or “heathy.” It is suggested that *Lactobacilli iners* was associated with CIN (23), or even CIN2^+^ (16).

We can infer from the above that these species could be used as microbiological markers of clinically significant disease. They can be used in the research for improved risk stratification of HPV-infected women who will ultimately develop cervical disease. We can find the cut-off relative abundance of potential microbiome biomarker by ROC Curve and explore the best scheme by running through every possible combination to help avoid unnecessary invasive cervical biopsy. For those infected with HPV type 16 and/or 18, the PPV of HSIL can reach from 76.19 to 82.54% when the relative abundance of *Stenotrophomonas* is over 0.0090387%, or *Faecalibacterium* under 0.01420015%, or *Bifidobacterium* under 0.0116183%. For those infected with the other 12 types of HR-HPV with concurrent abnormal TCT results, the PPV can reach from 40.00 to 67.50% when the relative abundance of *Stenotrophomonas* is over 0.01549105%, or *Streptococcus* over 0.48409585%, or *Bacteroides* under 0.0296912%. That is, 11 cases could avoid the invasive biopsy. This finding is of great importance for clinical practice.

The most outstanding strength of our study is the comprehensive comparison within those confirmed with HSIL, with high-risk HPV infection but without cervical lesions, and those without HPV infection. Also, we found the significantly different biomarkers assisting in predicting HSIL. However, there are also some limitations in this pilot study. Chronic genital inflammation may promote carcinogenesis similar to other mucosal sites. Although some clinical studies have revealed that HPV infection or clearance is not associated with increased levels of genital inflammation, one study showed increased levels of proinflammatory cytokines in patients with cervical dysplasia (25–27). This needs to be further explored. This pilot study is a cross-sectional study, thus our findings are focused on the association between vaginal microbiome and cervical neoplasm, not the causation.

## Conclusion

In conclusion, certain vaginal microorganism may be HPV-dependent cofactors for cervical neoplasia development. The potential microbial biomarkers play an important role in determining the risk of developing HSIL in women with HR-HPV infection and decrease unnecessary invasive examination. Future researches with continuous observation are required to confirm the association between dysbiosis of vaginal microbiota and HPV-induced cervical carcinogenicity.

## Data Availability Statement

The data generated for this study are deposited in the Genome Sequence Archive of National Genomics Data Center, link: https://bigd.big.ac.cn/gsa/browse/CRA004675, accession number is: CRA004675.

## Ethics Statement

The studies involving human participants were reviewed and approved by Ethics Committee of Peking Union Medical College Hospital (PUMCH), Beijing, China (No. JS-1634, registered on July 24, 2018). The patients/participants provided their written informed consent to participate in this study. Written informed consent was obtained from the individual(s) for the publication of any potentially identifiable images or data included in this article.

## Author Contributions

SW and JL developed the ideas for this study, designed and performed this scheme. XC, LW, XT, HS, and QF collected the samples and clinical data. The manuscript was drafted by XC and LW, revised by SW, and approved by all authors.

## Funding

This study was funded by National Key R&D Program of China (2017YFC1001200).

## Conflict of Interest

The authors declare that the research was conducted in the absence of any commercial or financial relationships that could be construed as a potential conflict of interest.

## Publisher's Note

All claims expressed in this article are solely those of the authors and do not necessarily represent those of their affiliated organizations, or those of the publisher, the editors and the reviewers. Any product that may be evaluated in this article, or claim that may be made by its manufacturer, is not guaranteed or endorsed by the publisher.
