# Supplementary material for: Research of the Potential Vaginal Microbiome Biomarkers for High-Grade Squamous Intraepithelial Lesion
Source: Front Med (Lausanne). 2021 Sep 21;8:565001. doi: 10.3389/fmed.2021.565001 (PMC8490638; doi:10.3389/fmed.2021.565001)
Supplement: Supplementary file 1 [file Data_Sheet_1.docx]

**Supplementary materials**

**DNA extraction and the amplification of bacterial 16S rRNA V4 gene region and Illumina sequence were showed in the supplementary material**

**1. DNA extraction.**

Total genomic DNA was extracted from cervical samples using CTAB method. 1000ul CTAB lysate was pipetted into 2.0ml EP tube, and 20ul lysozyme and then appropriate amount of sample was added to the lysate. The mixture was incubated for 1.5 h at 65 °C, during which the sample should be inverted several times to mix fully so that it can be fully lysed. After centrifugation, 950ul supernatant was mixed with equal volume phenol (PH 8): chloroform: isoamyl alcohol (25:24:1), and then centrifugated at 12000rpm for 10min. Supernatant was again mixed with equal volume chloroform: isoamyl alcohol (24:1), and centrifugated at 12000rpm for 10min. Supernatant was pipetted into 1.5ml centrifuge tube, and mixed with 3/4 volume of isopropanol, and precipitated at -20°C. After centrifuging, the liquid was washed with 1ml 75% ethanol twice, and then dried in ultra clean workbench or room temperature. The DNA sample was dissolved in 51 ul ddH2O, and hatched at 55-60 °C for 10min for solubilizing if necessary. 1ul RNase A was added to digest RNA, and the DNA sample was placed at 37°C for 15min. DNA concentration and purity was monitored on agarose gels at 1%. According to the concentration, DNA was diluted to 1ng/ul using sterile water.

**2. Amplification of bacterial 16S rRNA V4 gene region and Illumina sequence:**

Using extracted genome DNA as template, the V4 region of the bacterial 16S rRNA gene was PCR-amplified using the primers 515F (5’-GTGCCAGCMGCCGCGGTAA-3’) and 806R (5’-GGACTACHVGGGTWTCTAAT-3’) with the barcode. All PCR reactions were carried out with Phusion High-Fidelity PCR Master Mix with GC Buffer (New England Biolabs), using 25μl Taq PCR mix*2, 1μl 10uM Primer F, 1μl 10uM Primer FR, 2.5μl gDNA, 8.0μl H_2_O. An initial denaturation step of 95 °C for 5 min was carried out, followed by 34 cycles of denaturation (94 °C, 1 min), annealing (57 °C, 45 s) and extension (72 °C, 1 min), and a final elongation step of 10 min at 72 °C，cooling for 5 mine at 16 °C. Mix same volume of 1X loading buffer (contained SYB green) with PCR products and operate electrophoresis on 2% agarose gel for detection. Samples with bright main strip between 400-450bp were chosen for further experiments. PCR products was mixed in equidensity ratios. Then, mixture PCR products was purified with Qiagen Gel Extraction Kit (Qiagen, Germany). Sequencing libraries were generated using TruSeq® DNA PCR-Free Sample Preparation Kit (Illumina, USA) following manufacturer's recommendations and index codes were added. The library quality was assessed on the Qubit@ 2.0 Fluorometer (Thermo Scientific) and Agilent Bioanalyzer 2100 system. At last, the library was sequenced on an Illumina HiSeq. 2500 platform and 250 bp paired-end reads were generated.

**3. Data analysis.**

The original data were pretreated by BIPES data analysis process. In total 26,791,191 reads were obtained from 329 samples with an average number of reads per sample of 81,432 reads. Sequences analysis were performed by Uparse software (Uparse v7.0.1001). Sequences with a 97% similarity threshold were assigned to the same OUTs. Representative sequence for each OUT was screened for further annotation. Species annotation was analyzed using the Mothur method and the SSUrRNA database of SILVA (threshold value 0.8-1). Multiple sequence alignment were conducted using the MUSCLE software (Version 3.8.31) to study the phylogenetic relationship of different OTUs and the difference of the dominant species in two groups. The subsequent analysis include Alpha and Beta diversity analysis. Alpha diversity is applied in analyzing complexity of species diversity for a sample through 6 indices, including Observed-species, Chao 1, Shannon, Simpson, ACE, Good-coverage. All this indices in our samples were calculated with QIIME software (Version 1.7.0) and displayed with R software (Version 2.15.3). Chao 1 and ACE were selected to identify Community richness. Shannon and Simpson were used to identify Community diversity. And coverage was used to characterize Sequencing depth. Beta diversity analysis was used to evaluate differences of samples in species complexity. Based on the unweighted_unifrac distance, using QIIME software (Version 1.7.0) to carry out the analysis of principal component of vaginal microbiota. Cluster analysis was preceded by principal component analysis (PCA), which was applied to reduce the dimension of the original variables using the FactoMineR package and ggplot2 package in R software(Version 2.15.3). Principal Coordinate Analysis (PCoA) was performed to get principal coordinates and visualize from complex, multidimensional data. A distance matrix of weighted or unweighted unifrac among samples obtained before was transformed to a new set of orthogonal axes, by which the maximum variation factor is demonstrated by first principal coordinate, and the second maximum one by the second principal coordinate, and so on. PCoA analysis was displayed by WGCNA package, stat packages and ggplot2 package in R software(Version 2.15.3). Unweighted Pair-group Method with Arithmetic Means(UPGMA) Clustering was performed as a type of hierarchical clustering method to interpret the distance matrix using average linkage and was conducted by QIIME software (Version 1.7.0). Using linear discriminant analysis (LDA) coupled with effect size measurements (LEfSe) to analyze the difference of structure and composition of vaginal microbial communities between two groups.
